# Efficacy of oral irrigators compared to other interdental aids for managing peri-implant diseases: a systematic review

**DOI:** 10.1038/s41405-025-00301-3

**Published:** 2025-01-29

**Authors:** Gargi Gandhi, Bhoomika Sai Laxmi Masanam, Ananya Sudhakaran Nair, Nidhi Semani, Aditi Chopra, Venkitachalam Ramanarayanan

**Affiliations:** 1 Manipal College of Dental Sciences, Manipal, Manipal Academy of Higher Education, Manipal, Karnataka India; 2 Department of Periodontology, Manipal College of Dental Sciences, Manipal, Manipal Academy of Higher Education, Manipal, Karnataka India; 3 Department of Public Health Dentistry, Amrita School of Dentistry, Amrita Vishwa Vidyapeetham, Kochi, India

**Keywords:** Peri-implantitis, Dental implants

## Abstract

**Objective:**

Peri-implant diseases (peri-implant mucositis and peri-implantitis) are inflammatory conditions that affect the peri-implant tissues and are induced by microbial biofilms (dental plaque) formed around the implant. Removal of biofilm is the fundamental step in managing peri-implant diseases. Interdental cleaning aids such as interdental brush, unitufted brush, or oral irrigation along with regular toothbrushing are recommended for effective plaque control around implants. The present systematic review aims to evaluate the efficacy of home use of oral irrigators compared to other plaque control methods for managing peri-implant diseases.

**Method:**

This systematic review has been prepared using the Preferred Reporting Items for Systematic Review and Meta-analysis Protocols (PRISMA) checklist. Five databases were searched using the following keywords: “Peri-implantitis” OR Periimplantitis OR “Peri-implant disease” OR “Peri-implant Mucositis” AND “Oral irrigation” OR “Oral Irrigator” OR Waterpik OR “Interdental irrigation” OR “Oral Spray” OR “Oral Irrigants”.

**Results:**

Oral irrigator along with mechanical brushing was found to be more effective than mechanical brushing alone in reducing the plaque index (PI), gingival index (GI), and bleeding on probing (BOP). No statistically significant difference in improvement in quality of life and patient satisfaction with regard to comfort and ease of use was noted upon using mechanical toothbrushing alone and an oral irrigator. Implant sites where an oral irrigator was used showed more reduction in BOP (81.8% vs 33.35%) compared to sites where the floss was used (P = 0.0018). The levels of the red and orange complex bacteria in the peri-implant biofilm were lower with the use of an oral irrigator than with toothbrushing alone.

**Conclusion:**

Oral irrigators along with toothbrushing were found to be more effective in removing microbial plaque around implants and controlling peri-implant inflammation compared to mechanical brushing alone. The reduction in BOP was better with the use of oral irrigation compared to floss and interdental brush.

## Introduction

Peri-implant diseases, which include peri-implant mucositis and peri-implantitis, are one of the most common biological complications seen in patients with dental implants [1, 2]. The prevalence of peri-implantitis is rapidly increasing due to an increase in the replacement of teeth with dental implants [3, 4]. According to the 11th European Workshop in Periodontology in 2014, a patient-level prevalence of 43% for peri-implant mucositis and 22% for peri-implantitis was reported [5]. Another systematic review, including 47 studies, reported a prevalence similar prevalence of 46.83% for peri-implant mucositis and 19.83% for peri-implantitis [6, 7]. Peri-implant diseases are inflammatory in origin, caused primarily by the formation of peri-implant biofilms on the implant surface [8]. The peri-implant inflammation begins as an inflammation of the soft tissues surrounding the implant as peri-implant mucositis, which clinically manifests as swelling, color change, and bleeding from the peri-implant mucosa without any marginal bone loss [3, 9]. If left untreated, peri-implant mucositis progresses to peri-implantitis with the spread of inflammation to the underlying bone. Peri-implant mucositis manifests as bone loss, swelling, pocket formation, mobility of implant, and pus discharge from the peri-implant sulcus [10]. Progression of peri-implantitis will most likely lead to the loss of the affected implant and the implant-supported prosthesis.

Early diagnosis and management of peri-implant mucositis are crucial since it is a reversible condition. Addressing it promptly can help prevent the development of peri-implantitis. One of the primary risk factors for peri-implant diseases is inadequate maintenance and plaque control by patients, leading to biofilm formation on the implant surface and the surrounding mucosa [11, 12]. Adequate plaque control via effective periodontal debridement by dental professionals and patient education to maintain good oral hygiene at home are necessary for managing peri-implant diseases [11–14]. Toothbrushing along with interdental aids such as interdental, unitufted brushes, or oral irrigators are some of the common plaque control aids used by patients to maintain oral hygiene around dental implants [15, 16]. Gennai et al. [17] in their systematic review also emphasized the importance of home or patient-achieved plaque control measures along with professional debridement for the management of peri-implant mucositis [17]. The use of interdental aids is crucial, especially for patients with oral implants, because the lack of papilla between an implant and teeth or between two implants is bound to occur [18]. The toothbrush cannot completely remove biofilm from the interdental area and hence interdental aids for plaque removal from the interdental area are necessary. The interdental aids can be used alone or with antimicrobial or anti-inflammatory agents such as chlorhexidine, essential oils(Listerine®), saline, or Povidone-iodine [2, 4, 19]. Although the use of interdental aids to maintain effective plaque control is well-known, patients often do not use interdental aids regularly. Lack of motivation, and knowledge, reduced manual dexterity, and the need for additional time are common problems that preclude patients from using interdental aids. Hence, many clinicians recommend using oral irrigators instead of interdental brush or floss, as oral irrigators are a simpler, easier method for maintaining plaque control in patients with dental implants [15, 16].

An oral irrigator is a home care device that uses a pulsating or continuous stream of water at high pressure to flush or remove the biofilm and debris attached to the tooth or implant surface [20, 21]. Waterpik, Waterfloss, Aquapick, and Aquaflosser are examples of oral irrigators used for both in-office and at-home applications [22, 23]. Oral irrigators have proven to be effective adjuncts to toothbrushing for controlling gingival inflammation and biofilm formation for patients undergoing orthodontic treatment, those with dental prostheses, and dental implants [24–26]. Continuous use of oral irrigators can reduce bleeding on probing, pocket depth, and levels of pro-inflammatory mediators and pathogenic microbes. Numerous studies have compared the use of oral irrigators to other interdental aids such as floss, interdental brush, and toothpicks) for managing periodontal and peri-implant disease and provided mixed results [22–24, 27]. AlMoharib et al [27] conducted a meta-analysis and found that oral irrigators with mechanical brushing significantly improve the plaque scores and reduce bleeding scores compared to mechanical brushing alone in orthodontics patients with generalized gingivitis. No significant improvement in the gingival index was noted with the use of an oral irrigator along with mechanical toothbrushing compared to brushing alone [27]. For patients on periodontal maintenance, interdental brushes were better than oral irrigators for controlling plaque formation. However, when a similar comparison is made on gingivitis patients, a single use of the oral irrigator removed significantly more plaque than an interdental brush. Lyle et al [28] also noted that an oral irrigator (water flosser) was more effective than the interdental brush for removing plaque from all areas of the oral cavity. Water flosser was 18% more effective for removing plaque from the whole mouth and marginal areas, 20% for approximal areas, 11% for facial areas, and 29% for lingual areas after a single use [28]. A meta-analysis by Kotsaksi et al. also found that water-jet irrigation is more effective at reducing BOP than flossing [25]. On the contrary, Husseini et al. [26] found that the oral irrigator does not have any additional benefits in reducing plaque scores in patients with periodontitis [26]. Similarly, Slot et al in their network meta-analysis found low certainty of the evidence for the use of oral irrigators while there was moderate certainty for a small effect of the interdental brush as an adjunct to toothbrushing for managing gingival inflammation [29]. Interdental cleaning aids and interdental brushes were found to be better than oral irrigators [30].

The existing evidence explores the efficacy of oral irrigators for managing gingivitis and periodontitis, with limited reviews exploring the role of oral irrigators in managing peri-implant disease (peri-implant mucositis and peri-implant). Hence the present systematic review aims to explore the efficacy of oral irrigators at home compared to other mechanical plaque control methods for managing peri-implant diseases. With the marked rise in the use of dental implants for prosthetic rehabilitation, this review is timely and important for both the public and dentists. The review will help to understand the efficacy of oral irrigators compared to other plaque control methods for implant maintenance.

**Focus question**

How effective are home use of oral irrigators compared to other mechanical plaque control methods in managing peri-implant diseases?

## Objectives

### Primary objective

To evaluate and compare the efficacy of home use of oral irrigators compared to other mechanical plaque control methods (floss/ interdental brush) in reducing the BOP, gingival inflammation, probing pocket depth (PPD), and clinical attachment loss (CAL).

### Secondary objective

- To evaluate and compare the efficacy of oral irrigators compared to other mechanical plaque control methods in reducing biofilm formation and improving soft and hard tissue healing.
- To evaluate and compare patient satisfaction, improvement in quality of life, ease of use, and patient-reported adverse events/effects after the use of oral irrigators for managing peri-implant diseases.

## Methodology

This systematic review has been prepared using the Preferred Reporting Items for Systematic Review and Meta-analysis Protocols (PRISMA) checklist [31]. The protocol has been registered in the International Prospective Register of Systematic Review PROSPERO with registration number CRD42023469319 ON 14/10/2023. The study was under the exempt category of the ethical approval of the Institutional Ethics Committee of Kasturba Medical College and Kasturba Hospital, Manipal.

### Eligibility criteria

The eligibility criteria for screening the articles followed the Participant-Intervention-Comparison-Outcome-Study Design (PICOS) framework as follows:

- **Participants/condition:** Patients above 18 to 70 years with peri-implantitis or peri-implant mucositis in any type or design of dental implant placed in either maxilla or mandible were included.
- **Intervention:** Use of oral irrigators either with or without any chemical plaque control agent or mechanical toothbrushing to manage peri-implant mucositis or peri-implantitis.
- **Comparators:** Any other mechanical plaque control methods (mechanical toothbrush, dental floss, interdental brush) with or without any chemical plaque agents
- **Outcomes:** The following primary and secondary outcomes were reported:

***Primary outcome:*** PPD, CAL, gingival inflammation (measured by any gingival index (GI), bleeding on probing (BOP) as measured by any index, plaque scores (as measured by any plaque index (PI)).

***Secondary outcome:*** Bone loss around the implant; degree of osseointegration, recession around peri-implant mucosa, soft tissue healing (measured by any index), ease of use and patient satisfaction (measured by any index/ scale/ tool), cost-effectiveness, and improvement in quality of life (measured by any index/ scale/ tool) after using the oral irrigator.

- **Study design:** All non-randomized and randomized clinical studies, cross-sectional, prospective, case-control, and case series with more than 8 cases were included in this review. All case reports letters to the editor, narrative reviews, scoping reviews, and in-vitro/animal studies were excluded. No restrictions were placed on language or date of publication.

### Search strategy

Five databases (Medline (via PubMed), Scopus, Web of Science, EMBASE, and Cochrane along with the clinical trial registry (https://clinicaltrials.gov/) were searched electronically by four authors (GG, AN, BM, NS) using the following MeSH terms (“Peri-Implantitis”[Mesh]; “Oral Irrigator” “Endosseous Implants”) and Boolean operators as follows: “Peri-implantitis”[Title/Abstract] OR Periimplantitis[Title/Abstract] OR “Peri-implant disease”[Title/Abstract] OR “Peri-implant Mucositis”[Title/Abstract] AND “Oral irrigation”[Title/Abstract] OR “Oral Irrigator”[Title/Abstract] OR Waterpik [Title/Abstract] OR “Interdental irrigation”[Title/Abstract] OR “Oral Spray”[Title/Abstract] OR “Oral Irrigants”[Title/Abstract]. The preliminary search to check for the possibility of articles was done on 29-06-2023 and the final search was completed and updated on 8-2-2024. The search was complemented by hand searching of the reference list of included relevant articles. The corresponding authors for the ongoing clinical trials were requested to share the results (if available). All the articles were compiled in Microsoft Excel spreadsheet version 2308 and duplicates were removed.

### Data screening and data collection process

After removing duplicates manually in the Microsoft Excel spreadsheet, four authors (GG, AN, BM, NS) independently screened the studies using the above-mentioned eligibility criteria. In the first stage, the titles and abstracts screening for all the studies were independently done by four authors (GG, AN, BM, NS). Studies that did not qualify for the eligibility criteria and those with inadequate data or information were excluded. In the second stage, the four authors (GG, AN, BM, NS) independently screened the full texts of the included studies. Any disagreements regarding the inclusion of a study were discussed between the reviewers and, if unresolved, the subject expert (AC) was consulted and a final decision was made. Any articles whose full text was not available either online, in print, or even after requesting the corresponding authors were excluded. All studies that were selected after the full-text screening were reviewed by the supervisor (AC) before data extraction.

### Data item

The following data was collected from each study: author, year of study, site of study, study design, sample size, age range (mean age), male to female, type of peri-implant disease (peri-implant mucositis or peri-implantitis), nature of the intervention (Type of oral irrigator, frequency of use and nature and dose of chemical agent use with oral irrigator), Nature of the comparator (Type of mechanical plaque control agent, frequency, duration of use), Total no. of implants assessed, Type of implants in the study (Endosseous implants/ overdentures); Outcomes and method to assess the outcome (mean score ± standard deviation for GI, Plaque score, BOP, PPD, CAL, bone levels, Microbial profile, inflammatory biomarker); Patient-reported outcomes (ease of use, adverse events or problem reported while using oral irrigators).

### Risk of bias assessment

The risk of bias was assessed using the revised Cochrane Risk of Bias Assessment Tool (ROB2) [32]. The following domains were assessed for risk of bias: 1. Randomization process; 2. Bias resulting from deviations from intended interventions; 3. Bias resulting from missing outcome data; 4. Bias resulting from measurement of the outcome; 5. Bias resulting from selection of the reported result; 6. Bias in the identification or recruitment of individual participants within clusters. The identified risk was categorized as low risk, high risk, and risk with some concern. The ‘effects of assignment’ were considered as the effect of principal interest to assess the risk of bias. The quality assessment of non-randomized studies was performed using the ‘Risk of Bias in Non-Randomized Studies of Interventions (ROBINS-I)’ tools. The following seven domains were used for Risk of Bias analysis: a) bias due to confounding; b) bias in the selection of participants into the study; c) bias in the classification of interventions; d) bias due to deviations from intended interventions; e) bias due to missing data; f) bias in the measurement of outcomes; g) bias in the selection of the reported result. The scores equal to or less than 4 were considered a high risk of bias; scores between 5-7 were considered a moderate risk of bias and scores above than or equal to 8 were considered a low risk of bias.

### Assessment of heterogeneity and reporting bias

Clinical and methodological heterogeneity was assessed by comparing the specifications of intervention and control used, outcome measures, and measurement tools used in each included study.

### Effect measures and data synthesis

Data synthesis was performed by qualitatively summarizing and comparing each included article for its study characteristics: study population, intervention and controls, outcome measures, tools of measurement, and results. The mean and standard deviation for PI, GI, BOP, PD, CAL, Microbial count, and levels of biomarkers were noted along with the P-value. A Summary of Findings (SoF) for narrative reviews was prepared using GradeProGDT. The SoF has been prepared to identify the effectiveness of oral irrigators in reducing the PPD, plaque index, gingival index, and BOP.

## Results

### Studies included

**Fig. 1:**
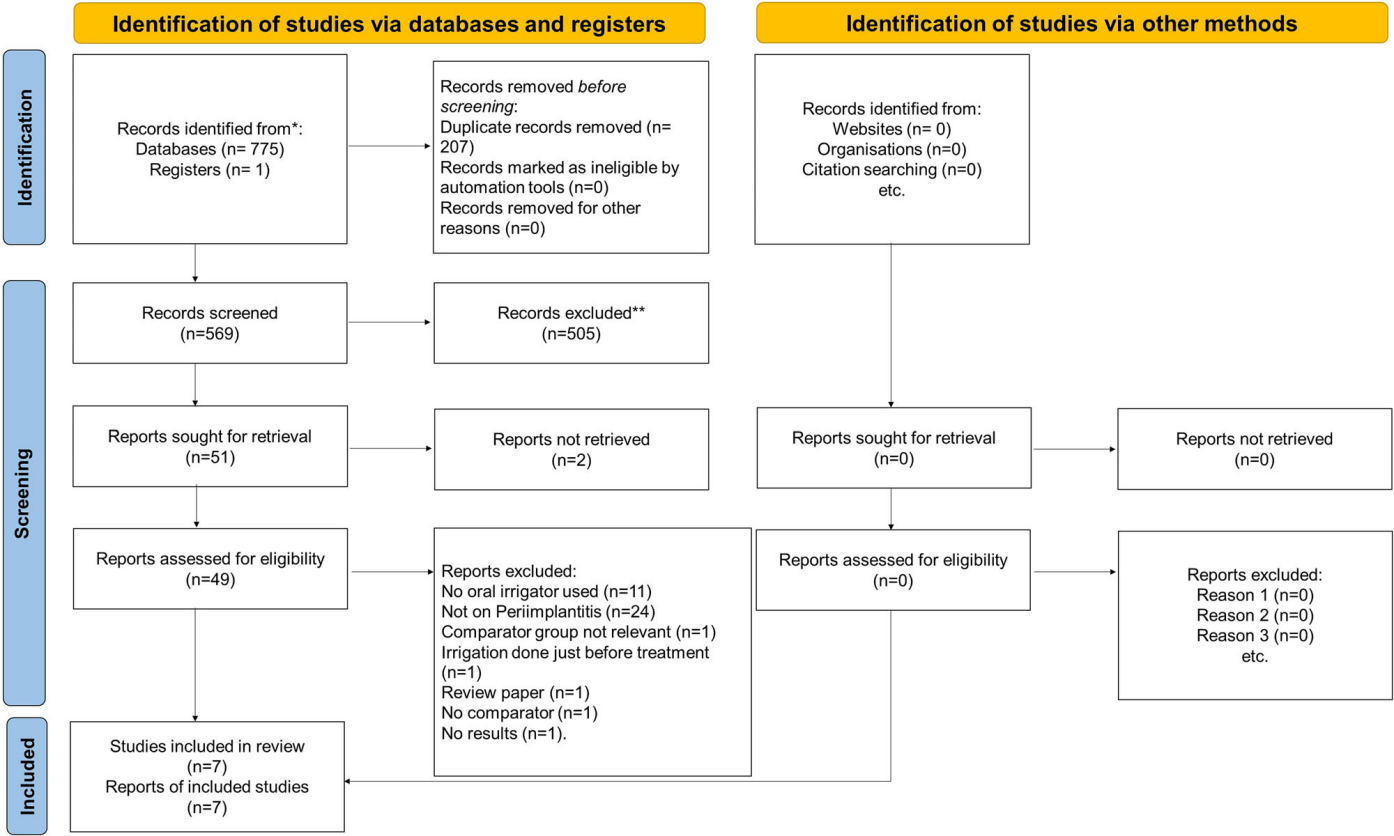
Prima flow diagram.

The search strategy identified a total of 776 articles. After the removal of duplicates (N = 207), 569 articles were included for the title and abstract screening. Out of 569 articles, 49 articles were taken up for full-text screening. Out of 49, 42 articles were excluded as they did not meet the eligibility criteria (PICO). The detailed list of excluded studies with reasons for exclusion is provided in the supplementary Table 1. After complete full-text screening, a total of seven articles were included in our systemic review for data extraction and quantitative synthesis (Fig. 1: PRISMA flow diagram) [33–42]. The detailed characteristics of the included studies are summarized in Table 2 and Table 3.

### Study characteristics

Of all the seven included studies, two studies were from Brazil [43, 44], one from Germany [45], one study from Uzbekistan [46], one from the USA [47], one from Canada [48], one from Turkey [33], 2022). A total of 360 participants were included in this review. The sample sizes ranged from 28 to 92 participants [46, 47]. The age range of the participants was from 22 to 89 years [45, 47]. There were two cross-over studies [34, 43]; and five parallel-arm randomized studies [33, 45–48]. The following methods of randomization were noted: block randomization [33, 38]; tables of random numbers [46]; computer-generated list [33, 34, 43, 48], and random sequencing generated using SPSS software [41]. Five studies were single-blinded [33, 34, 43, 47, 48] and two studies were non-blinded studies [45, 46]. The follow-up ranged from 14 days to 6 months (Table 1) [34, 43, 46–48]. The total implants analyzed ranged from 38 to 76. The oral irrigators were Waterpik [34, 43, 45–48] and Oxyjet Oral Irrigator [33]. The oral irrigator was used along with mechanical tooth brushing either once [33, 34, 43, 45, 47, 48] or twice before or after brushing [46]. One study by Magnsuson et al. recommended the use of Waterpik before toothbrushing, other studies educated their participants to the use of an oral irrigator after toothbrushing [47]. Oral irrigation was done either with plain water [34, 43, 45]; tap water [48]; warm water [33, 47]; and Chlorhexidine (0.06%) [45].

**Table 1:** Detailed list of articles retrieved from different databases and search strings used for data collection.

Database Searched	Database provider	Date and Person searched	Keywords/ Strategy	No. of studies
MEDLINE	PubMed	29/06/23	“Peri-implantitis”[Title/Abstract] OR Periimplantitis [Title/Abstract] OR “Peri-implant disease”[Title/Abstract] OR “Peri-implant Mucositis”[Title/Abstract] AND “Oral irrigation”[Title/Abstract] OR “Oral Irrigator”[Title/Abstract] OR Waterpik[Title/Abstract] OR “Interdental irrigation”[Title/Abstract] OR “Oral Spray”[Title/Abstract] OR “Oral Irrigants”[Title/Abstract]	253
SCOPUS	Elsevier	29/06/23	(TITLE-ABS-KEY (periimplantitis) OR TITLE-ABS-KEY (“peri-implantitis”) OR TITLE-ABS-KEY (“peri-implant disease”) OR TITLE-ABS-KEY (“peri-implant mucositis”) AND TITLE-ABS-KEY (“oral irrigation”) OR TITLE-ABS-KEY (“oral irrigator”) OR TITLE-ABS-KEY (Waterpik) OR TITLE-ABS-KEY (“interdental irrigation”) OR TITLE-ABS-KEY (“oral spray”) OR TITLE-ABS-KEY (“oral irrigant”))	4
Cochrane		29/06/23	Title abstract keywords (oral irrigators and periimplantitis)	11
Web of Science	Clarivate	29/06/23	(TI= (‘periimplantitis’ OR ‘periimplantitis’ OR ‘peri-implant disease’ OR ‘peri-implant mucositis’)) AND (TI = (‘oral irrigation’ OR ‘oral irrigator’ OR ‘Waterpik’ OR ‘interdental irrigation’ OR ‘oral spray’)))	3
EMBASE	Elsevier	29/06/23	(‘periimplantitis’ OR ‘periimplantitis’ OR periimplantitis OR ‘peri-implant disease’ OR ‘peri-implant mucositis’ OR ‘peri-implant mucositis’) AND ‘oral irrigation’ OR ‘oral irrigator’ OR ‘Waterpik’ OR ‘interdental irrigation’ OR ‘oral spray’/exp OR ‘oral spray’ OR ‘oral irrigants’	505
			**Total**	**776**
			**Duplicates**	**207**

The following clinical parameters were assessed in the review: modified PI (Mombelli et al. [49]) [34, 43, 45]; Full mouth PI by Quigley Hein [48]; GI by Loe [50] [34, 43]; BOP by (Dichotomous index) [34, 43, 45, 48], ‘Carter and Barnes’ bleeding index and percentage of the site with BOP [47] and mucositis severity scores [45]; probing depth [34, 43, 48] and width of keratinized tissues [48]. Only one study checked the changes in the microbial profile [43]. Only one study reported the changes in the proinflammatory cytokines in the gingival crevicular fluid (GCF) around the implant after the use of oral irrigators [33]. The patient’s satisfaction score, ease of use, and compliance outcomes/satisfaction scores were assessed by two studies [43, 48]. Five studies compared the use of oral irrigators (Waterpik) with mechanical brushing alone [33, 34, 43, 45, 46]. Two studies compared the use of oral irrigators with interdental brushes [33, 46] and two studies compared the efficacy of oral irrigators to dental floss [47, 48]. One study by Bunk et al. [45] used a specialized patient education tool (CIOTI-plus), along with brushing technique and oral irrigation [45]. All studies were conducted using single-endosseous implants except two studies by Salles et al. [43] where the use of an oral irrigator was assessed for implant-supported overdenture [34, 43]. The changes in the marginal bone levels, degree of osseointegration, and radiographic bone changes were not reported by any study.

### Quality (risk of bias) assessment

Four studies were found to have a low risk of bias [33, 34, 43, 45]; one study had some concerns [47] and two studies had a high risk of bias [46, 48]. The main concern was that no baseline data was provided for the clinical parameter (gingival index) in one study [46]. The uncertainty in the randomization process and blinding of the patient and investigator was noted in all the studies. The self-administered questionnaire which was developed by a qualified committee is also not specified. Salles et al did not state whether patients with peri-implantitis and peri-implant mucositis or both were included in the inclusion criteria. In the study by Azim and Artur, the reduction in plaque accumulation was assessed by the patients themselves, which is of concern as to how the patient differentiates between debris, plaque, and calculus (Supplementary Table 2).

**Table 2:** Characteristics of the included studies.

Authors/ Year	Study location	Study Design (N= sample size)	Age Range Mean Age	Follow-up	Gender Ratio (Males: Females)	Intervention (Type of oral irrigator, frequency, and dose) Adjunct- Oral irrigators used with water, saline, or CHX (N=sample size)	Comparator (Type, frequency, dose) (N=sample size)	Total no. of implants	Type of implants (Endosseous implants/ overdentures)	Outcomes (mention only indices) and Methods to measure outcomes (Type of periodontal probe used)
Magnuson, et al. [48]	USA	Single-blind, double parallel-arm single-centered randomized clinical trial (N = 28)	22–62 years	0→ 14 days→ 30 days	Not mentioned	Waterpik, once daily before brushing. Adjunct- Luke warm water N = 15; mean age: 49.6 ± 10.6 years)	Unflavored waxed string dental floss (Reach®)- used before brushing in the evening. N = 13; mean age: 47.8 ± 12.0)	40	Not mentioned	Percentage of sites with BOP (six sites checked for each tooth using a plastic probe).
Salles et al. [45]	Brazil	Randomized crossover clinical study (N = 38)	Mean age: 60.8 ± 67.1 years.	0→ 14 days	9:29	WaterPik once a day after their last daily brushing (Oral B indicator brush) for 20 secs for each implant. Adjunct- water (N = 38)	Mechanical brushing 3 times a day in horizontal motion via a soft bristle brush for 2 min (N = 38)	Implant and overdentures (numbers not mentioned)	Maxillary conventional complete dentures and mandibular overdentures retained by two to four implants (O-ring-retained system)	Modified PI, GI, PD, and BOP (present/ absent); questionnaire using a visual analog scale to measure patient satisfaction; level of comfort after use; and feeling of cleanliness of the implants and prostheses; ease of daily use; continuation of use; and difficulties or complaints regarding the use (Colorvue, Hu-Friedy Co, Chicago, Ill)
Olimov et al [46]	Uzbekistan	Randomized non-blinded parallel arm single-centered study (N = 92)	Not mentioned	0→1month→ 3months→ 6 months	Not mentioned	Waterpik WP-660 (Aquarius) + brushing twice per day for 3–5 min + with Interdental brush (N = 45) Adjunct- water (type not specified).	One comparator group used a manual toothbrush (N = 13) and the second comparator group used manual brushing + Interdental brush (N = 30).	Not mentioned	Implant-supported crowns	OHI-S; GI (Loe & Silness [56])
Bunk et al. [46]	Germany	Single-blinded and single-centered parallel arm randomized clinical trial (N = 60)	70 (25–89) years	0→ 4 weeks→8 weeks→ 12 weeks	27:30	Oral irrigator (Waterpik® Cordless Plus Water Flosser WP-450) with plain water (Group 1: N = 20; mean age: 68.5 (25–83 years)) or with 0.06% CHX (group 2: N = 20; mean age: 70 (52–81)) once a day with 50 ml of the respective solution after toothbrushing and interproximal cleaning in the evenings	Toothbrushes alone (Curaden Germany GmbH, Curaprox CS 5460) and fluoride-containing toothpaste (Procter & Gamble GmbH. (N = 20; mean age: 71.0 (25–89 years))	60	Not mentioned	PD, BOP (% of sites), mucositis severity score (Grischke et al. [57]), modified GI (Löe, [50]), Modified PI for dental implants (Mombelli et al [49])
Tütüncüoğlu et al. [34]	Turkey	Randomized, single-blinded, parallel designed (N = 45)	45-60	0→2 weeks→4 weeks→12 weeks	20:25	Oral irrigator (Oxyjet Oral Irrigator, Germany) once daily after brushing in the evening with a toothbrush (Oral-BR Soft Compact 35, Procter & Gamble, Germany) for 2 mins Adjunct- warm distilled water (N = 15; mean age: 53.93 ± 4.57)	Group 1: Interdental brush (Oral-B® Pro-Expert Clinic Line Interdental starter kit, Germany-B®) in the evening only after brushing (N = 15; mean age: 54.80 ± 4.60). Group 2: Toothbrush (Oral-B® Soft Compact 35, Procter & Gamble, Gross-Gerau, Germany) alone twice a day for 2 mins with modified bass technique. (N = 15; mean age: 52.73 ± 5.27)	Not mentioned	Implants (Straumann, Waldenburg, Switzerland) functioning at least 24 months before the study.	Modified PI, mSBI, PD, CAL, and BOP. PPD and PAL measurements were conducted using an automated periodontal probe (Florida Probe). Levels of interleukin 1 beta (IL-1β), transforming growth factor-beta (TGF-β), tissue-type plasminogen activator (t-PA), and plasminogen in the peri-implant crevicular fluid (PICF)
Sgarbanti et al. [51]	Canada	Single-centered, parallel arm single-blinded Randomized clinical study (N = 24)	48 - 85 years Mean age: 68 years	0→ 3months→ 6 months	17:16	Waterpik Water Flosser (WP-600, USA) once daily for 30 s. Adjunct- tap water (N = 12)	Dental floss (TePe Bridge and Implant Floss, Sweden) once daily for 12 months at night (N = 12)	76	Implant with a screw-retained crown.	PI-Quigley-Hein PI, PD, BOP- UNC 12 Colorvue probe, the width of the keratinized tissue (KT); questionnaire for liked interproximal device and ease of use (rating scale 1 to 5)
Salles et al. [44]	Brazil	Randomized cross-over single-centered clinical study (N = 38)	Mean age: 57.9 ± 6.2	0→14 days (7 days washout period)	9:29	WaterPik once a day after their last daily brushing for 20 s for each implant in the evening after brushing Adjunct- plain water (N = 38)	Mechanical brushing with a a soft bristle toothbrush (Oral B Indicator Plus – Procter & Gamble) n horizontal motion for 2 min, 3 times a day (N = 38)	Not mentioned	Maxillary conventional overdentures complete dentures and mandibular retained by 2 to 4 implants, with an O-ring-retained system.	Number of microbial species colonizing the subgingival sulci and overdentures using the checkerboard DNA–DNA hybridization technique (34 bacterial and 5 fungal species)

*PI* Plaque index, *GI* Gingival index, *PD* Probing depth, *BOP* Bleeding on probing, *CAL* Clinical attachment loss, *Sec* seconds, *N* number, *OHI-S* Simplified Oral Hygiene Index, *mSBI* Modified sulcus bleeding index

**Table 3:** Quantitative data for Oral hygiene status, plaque scores, Gingival Index, Bleeding on Probing, Mucositis severity index, Periodontal probing Depth, Clinical attachment Loss, Microbial profile, Width of keratinized tissue, Systemic effect (levels of inflammatory mediator, patient satisfaction score for all the included studies).

Authors et al., Year	Magnuson et al. [48]	Olimov et al [46]	Bunk et al. [46]	Salles et al. [45]	Salles et al. [45]	Sgarbanti et al. [51]	Tütüncüoğlu et al. [34]
Groups/ variables	OR and Dental floss	OR + MB; MB + IDB and MB alone	OR with plain water + MB; OR with 0.06% CHX + MB and MB alone	OR and MB	OR and MB	OR with water and Dental floss	OR warm distilled water + MB; IB + MB; MB
**Oral hygiene status (PI/Debris index/OHI-S/FMPS)**	NR	**OHI-S** Baseline not mentioned OR - 1 month: 0.6; 3 months: 0.6; 6 months: 1.0 MB + IDB – 1 month: 0.6; 3 months: 0.8 6 months: 1.6 MB alone: 1 month: 0.7; 3 months: 1.2; 6 months: 1.7 **P-value:** NR	**Modified PI** Baseline OR+ Water+ MB: 1.19 ± 0.49 OR + CHX + MB: 1.26 ± 0.40 MB alone: 1.33 ± 0.52 Four weeks OR+ Water+ MB- 1.00 ± 0.67 OR + CHX + MB- 1.03 ± 0.60 MB alone- 1.06 ± 0.43 Eight weeks OR+ Water+ MB- 0.61 ± 0.53 OR + CHX + MB- 0.80 ± 0.56 MB alone- 0.98 ± 0.54 12 weeks OR+ Water+ MB- 0.79 ± 0.60 OR + CHX + MB- 0.75 ± 0.58 MB alone- 0.83 ± 0.63 **P-value: 0**	**Modified PI** Baseline – OR:2.64; MB: 2.64 14th day- OR: 1.66; MB: 1.70 **P-value:** < **0.001**	NR	**PI-Quigley-Hein PI** Baseline Floss: 0.70 OR: 0.47 Three months Floss: 0.65 OR: 0.31 Six months Floss: 0.58 OR: 0.42 P-value: 0.14 **FMPS** Baseline Floss: 40.08 OR: 42.50 Three months Floss: 33.92 OR: 38.75 Six months Floss: 16.50 OR: 27.33 **P-value: 0.57**	**Modified PI** Baseline OR + MB: 1.73 ± 0.46 IB + MB: 1.80 ± 0.41 MB: 1.67 ± 0.49 Two weeks OR + MB: 1.00 ± 0.38 IB + MB: 1.33 ± 0.35 MB: 1.07 ± 0.26 Four weeks OR + MB: 0.33 ± 0.49 IB + MB: 0.67 ± 0.49 MB: 1.00 ± 0.00 12 weeks OR + MB: 0.33 ± 0.49 IB + MB: 0.67 ± 0.26 MB: 0.67 ± 0.49 **P** < **0.05**
**Gingival Index**	NR	**Modified GI (Loe et al.** [50]**)** OR + MB: No quantitative value mentioned. After 6 months, it significantly differed from the control group by 92%. MB + IDB: baseline: NR; 3 months: 0.57 ± 0.05; 6 months: 1.2 ± 0.05. MB: baseline: NR; 6 months: 1.7 ± 0.05	NR	**Modified GI (Loe et al.** [50]**)** Baseline- OR:2.75 MB: 2.75 14th day- OR:1.61; MB: 1.67 **p-value:** < **0.001**	NR	NR	NR
**Bleeding on Probing (BOP)**	**Presence/absence of BOP** OR: No of sites with BOP Baseline 18 (100%); Day 14: 9 (50%); Day 30: 12 (33.3%) **Floss** Baseline: 22 (100%) Day 14 - 5 (77.3%) Day 30: 4 (81.8%) **P** = **0.0018**	NR	**Presence/absence of BOP** Baseline MB with Instructions only: 2.35 ± 0.99; OR (water)+MB: 2.25 ± 1.02 OR (CHX) + MB: 2.40 ± 0.88 Four weeks MB with Instructions only: 1.20 ± 0.77 OR (water)+MB: 1.15 ± 1.18 OR (CHX) + MB: 0.75 ± 0.97 8 weeks MB with Instructions only: 1.00 ± 1.03 OR (water)+MB: 0.80 ± 1.06 OR (CHX) + MB: 0.75 ± 1.07 12 weeks MB with Instructions only: 0.85 ± 1.09 OR (water)+MB: 0.45 ± 0.69 OR (CHX) + MB: 0.10 ± 0.45 Water Irrigation vs Instructions only p-value: 0.12 CHX irrigation vs Instructions Only: P-value: 0.004 CHX irrigation vs Water irrigation: P-value: 0.16	**Presence/ absence of BOP** Baseline - OR: 2.54; MB: 2.54 14^th^ day- OR: 1.61 MB: 1.86 (**P** < **0.001)**	NR	**Presence/absence of BOP** Baseline Floss: 7.52 OR: 14.82 Three months Floss: 9.68 OR: 8.83 Six months Floss: 6.67 OR: 12.25 **P-value: 0.77**	**mSBI** Baseline OR + MB: 2.00 ± 0.00 IB + MB: 2.00 ± 0.00 MB: 2.00 ± 0.00 Two weeks OR + MB: 0.80 ± 0.41 IB + MB: 1.27 ± 0.59 MB: 1.53 ± 0.52 Four weeks OR + MB: 0.07 ± 0.26 IB + MB: 0.6 ± 0.51 MB: 1.13 ± 0.35 12 weeks OR + MB: 0.00 ± 0.00 IB + MB: 0.33 ± 0.49 MB: 0.87 ± 0.64 *(p* > 0.05) **% of sites with BOP** Baseline OR + MB: 1.00 ± 0.00 IB + MB: 1.00 ± 0.00 MB: 0.93 ± 0.26 Two weeks OR + MB: 0.47 ± 0.52 IB + MB: 0.93 ± 0.26 MB: 0.87 ± 0.35 Four weeks OR + MB: 0.00 ± 0.00 IB + MB: 0.27 ± 0.46 MB: 0.8 ± 0.42 12 weeks OR + MB: 0.00 ± 0.00 IB + MB: 0.00 ± 0.00 MB: 0.47 ± 0.52 (P > 0.05)
**Mucositis severity index**	NR	NR	Baseline OR+ Water+ MB: 9.00 ± 2.03 OR + CHX + MB: 9.05 ± 1.79 MB alone- 9.05 ± 2.54 Four weeks OR+ Water+ MB: 5.90 ± 2.95 OR + CHX + MB: 5.20 ± 3.43 MB alone: 6.30 ± 2.11 Eight weeks OR+ Water+ MB: 4.45 ± 3.85 OR + CHX + MB: 4.10 ± 3.04 MB alone: 5.25 ± 2.75 12 weeks OR+ Water+ MB:2.80 2.84 OR + CHX + MB: 2.10 ± 2.22 MB alone: 4.50 3.27 A significant improvement in the severity of peri-implant mucositis with CHX irrigation compared to oral hygiene instructions only (−2.4 [95% CI − 4.19; −0.61], p =.001). The use of an irrigation device with water compared to the control resulted in an estimated drop of 1.7 points in mucositis severity score after 12 weeks close to the significance threshold ( − 1.7 [95% CI − 3.49; 0.1], p = 0.06)	NR	NR	NR	NR
**Periodontal probing Depth (PD)**	NR	NR	Baseline OR+ Water+ MB- 3.3 mm OR + CHX + MB- 3.5 mm MB alone- 3.3 mm Four weeks Eight weeks 12 weeks Probing depth not mentioned P-value: not mentioned	Baseline – OR: 2.62; MB: 2.62 14 days: OR: 1.61; MB: 1.78 (P < 0.001)	NR	Baseline OR: 3.61 Floss: 3.31 Three months OR: 2.94 Floss: 3.28 Six months OR: 3.08 Floss: 2.76 p-value (0.39)	Baseline OR + MB: 2.53 ± 0.52 IB + MB: 2.53 ± 0.52 MB: 2.33 ± 0.49 Two weeks OR + MB: 2.53 ± 0.52 IB + MB: 2.47 ± 0.52 MB: 2.33 ± 0.49 Four weeks OR + MB: 2.53 ± 0.52 IB + MB: 2.47 ± 0.52 MB: 2.33 ± 0.49 12 weeks OR + MB: 2.53 ± 0.52 IB + MB: 2.47 ± 0.52 MB: 2.33 ± 0.49 *(p* > 0.05)
**Clinical Attachment Loss (CAL)**	NR	NR	NR	NR	NR	NR	Baseline OR + MB: 2.53 ± 0.52 IB + MB: 2.53 ± 0.52 MB: 2.33 ± 0.49 Two weeks OR + MB: 2.60 ± 0.63 IB + MB: 2.53 ± 0.52 MB: 2.27 ± 0.59 Four weeks OR + MB: 2.53 ± 0.52 IB + MB: 2.47 ± 0.52 MB: 2.27 ± 0.59 12 weeks OR + MB: 2.53 ± 0.52 IB + MB: 2.47 ± 0.52 MB: 2.27 ± 0.50 *(p* > 0.05)
**Microbial profile**	NR	NR	NR	NR	Baseline: Red complex: 4.75 ± 6.01; Orange complex: 4.03 ± 4.50; Green complex: 3.85 ± 3.62; Purple complex 2.57 ± 3.17. Total microbial load: 27.70 ± 20.42 MB group after 14 days Red complex: 4.52 ± 4.68; Orange complex: 5.56 ± 5.37; Green complex: 4.42 ± 4.40; Purple complex: 2.15 ± 3.23; Total microbial load: 31.90 ± 25.71 OR group after 14 days Red complex: 3.72 ± 4.25; Orange complex: 3.98 ± 4.98; Green complex: 2.66 ± 2.87; Purple complex: 1.24 ± 2.73^c^; Total microbial load: 22.47 ± 19.57; P-value: Red complex: 0.749; Orange complex: < 0.001; Green complex: 0.030; Purple complex: 0.012; Total microbial load: 0.007	NR	NR
**Width of keratinized tissue**	NR	NR	NR	NR	NR	Baseline Floss: 1.98 OR: 2.29 Three months Floss: 2.02 OR: 2.33 Six months Floss: 2.19 OR: 2.17 P-value: 0.41	NR
**Systemic effect (levels of inflammatory mediator)**	NR	NR	NR	NR	NR	NR	A statistically significant differences were found in the IL-1β total volume between the OI and C groups only at the 12th week (*p* = 0.013), while there were no significant differences in this parameter between other groups for IL-1β, TGF-β at any other time points (*p* > 0.05). When the T-PA total values are analyzed, the results in the OI group were significantly lower than those in the C group at the 2nd (*p* = 0.003), 4^th^ (*p* = 0.015), and 12th week (*p* < 0.001) evaluations. The PAI-1 total values showed a statistically significant difference between the OI group and the C group at the 4^th^ (*p* = 0.011) and 12th weeks (*p* = 0.002). Test group comparisons showed that the PAI-1 total values in the OI group were significantly lower compared with those in the IB group in the 2nd week (*p* < 0.001)
**Patient satisfaction and score**	NR	NR	NR	Comfort after using the method- Z: - 1.609; p-value: 0.108 Cleansing sensation around the implants after using the method -Z: -1.540; p-value: 0.123 Cleansing sensation of overdenture after using the method -Z: -0.022; p-value:0.983 Ease of use in the daily hygiene of implants - Z: - 0.432; p-value:0.665 Ease of use in the daily hygiene of overdentures - Z: -0.402; p-value: 0.687 Level of satisfaction after using the method on implants – Z: -0.504; p-value:0.614 Level of satisfaction after using the method on overdentures Z: - 0.098; p-value:0.922 Continuity of use in daily hygiene -Z: -0.196; p-value: 0.845 Indicating the use of the method to friends and relatives-Z: -0.358; p-value: 0.721	NR	Questionnaire rating (1-5) Liked interproximal device: OR: 4.25 Floss: 3.75 Sig:.53 Ease of use OR: 3.42 Floss: 3.83 Sig: 0.41	
**Adverse Event**	NR	NR	NR	5 patients complained that gums around the implants were painful soon after using the OR and they found the OR was difficult to use.	NR	NR	NR

*OR* oral irrigator, *MB* Mechanical brushing, *IDB* Interdental Brush, *NR* not reported, *PI* Plaque Index, *DI* Debris index, *OHI* oral hygiene index, *FMPS* Full Mouth Plaque Score, *HI* Hygiene Index, *PD* periodontal probing depth, *CAL* Clinical attachment Loss, *NR* not mentioned

^a^Interleukin 1 beta (IL-1β), transforming growth factor-beta (TGF-β), tissue-type plasminogen activator (t-PA), Peri-implant crevicular fluid (PICF).

^b^Complex bacterial counts smaller than mechanical brushing.

^c^Complex bacterial counts smaller than baseline.

### Results from individual studies (Table 2 and Table 3)

Oral irrigator along with mechanical brushing was found to be more effective in reducing the PI, GI, and BOP than the use of oral irrigator alone or mechanical brushing alone [43] (Table 2). Oral irrigator when paired with a manual toothbrush was found to be 2.45-fold (145%) more effective than floss in reducing bleeding around implants. Implant sites where an oral irrigator (water flosser) was used showed more reduction in BOP (81.8% vs 33.35%) compared to sites where the floss was used (P = 0.0018) [47, 48]. The use of oral irrigators was effective in increasing the width of keratinized gingiva around implants and the increase in keratinized gingiva was found to be comparable to the floss (oral irrigator: 2.02 ± 2.33 mm and floss: 2.19 ± 2.17 mm) (Table 2) [48]. When oral irrigators were combined with a manual toothbrush and interdental brush, the reduction in PI and GI was better compared to the use of interdental brush and oral irrigator alone (Table 2). The control of gingival inflammation was more for patients who only used mechanical toothbrushes (1.7 ± 0.05) compared to those who used interdental brushes along with toothbrushes (0.95 ± 0.05) and oral irrigators along with interdental brushes and toothbrush (0.08) [46]. The PI and BOP were also significantly lower in the oral irrigator group compared with the toothbrush alone group (p < 0.05). No significant differences were noted for BOP scores for oral irrigator, mechanical brushing, and interdental brush at the end of 12 weeks (p > 0.05). Oral irrigators are also found to be effective in reducing the levels of proinflammatory cytokines (IL-I β and T-PA) compared to the toothbrushing group (p < 0.001) [33]. This evidence is low as only one study has reported the changes in the levels of the proinflammatory cytokine around implants with the use of oral irrigators.

The efficacy of oral irrigators is improved when used along with antimicrobial agents such as chlorhexidine. The use of an oral irrigator along with 0.06% chlorhexidine was found to be more effective compared to plain/ tap water as an adjunct to toothbrushing for managing peri-implant mucositis. The prevalence of peri-implant mucositis was found to be 5% when an oral irrigator was used with 0.06% chlorhexidine compared to 35% with plain water and 50% for toothbrushing alone. Upon comparison between the use of plain water and chlorhexidine, less reduction in BOP with water was noted compared to the use of chlorhexidine (Table 2). The patients who used chlorhexidine along with oral irrigation showed significantly lower BOP-positive sites after 12 weeks when compared to those who used toothbrushing alone (−0.75 [95% CI − 1.26; −0.25], p = 0.004) [45].

Oral irrigators were also found to be effective in reducing the microbial load compared to tooth brushing. Only one study that found changes in the levels of the microbial complexes (orange (P < 0.001), purple (P = 0.012), and green (P = 0.030) complexes) were more with the use of an oral irrigator compared to toothbrushing alone in patients with implant-supported overdentures [34, 43]. A reduction in the levels of *Campylobacter rectus* (P = 0.001), V*eillonella parvula* (P = 0.012), and *Porphyromonas endodontalis* (P = 0.003) was noted after the use of an oral irrigator compared to the toothbrush alone. No pathogenic bacteria like *Aggregatibacter actinomycetemcomitans, Bacteroides fragilis, Enterococcus faecalis, Lactobacillus casei, Staphylococcus pasteuri, Streptococcus constellatus, Streptococcus mutans* were not identified in the biofilm after the use of oral irrigator. (Table 2). No study was found to report any changes in the bone level after the use of an oral irrigator.

When assessing the patient-reported outcomes and ease of use of oral irrigators compared to other mechanical plaque control agents, no statistically significant differences in the ease of use and preference for using an oral irrigator or floss were reported by patients with dental implants No statistically significant difference in improvement in quality of life and patient satisfaction in patients using mechanical toothbrushing alone and oral irrigator with regards to comfort (P = 0.108); cleaning sensation around implants (P = 0.123), and overdentures (P = 0.983). No difference was noted concerning the ease of use by patients with endosseous implants (P = 0.665) and for those with overdentures (P = 0.687). No difference was noted in the ease of use and cleaning efficacy between the use of an oral irrigator and toothbrush (for cleansing sensation: Z = −1.540; P = 0.123) [34, 43]. No difference was noted in the level of satisfaction and cleanliness around implants (P = 0.614), overdentures (P = 0.922), and continuity of use in daily hygiene (P = 0.845). Only, one study reported the presence of pain in the gums and difficulty using oral irrigators by five patients.

### Results syntheses

A quantitative synthesis was not performed due to wide heterogeneity among studies in terms of intervention and comparator groups. The clinical heterogeneity was noted in terms of variations in the characteristics of the study population and outcome assessed. For example, a study by Bunk et al. mentions that both periimplantitis and peri-implant mucositis patients were included; while other studies did not differentiate between periimplantitis and peri-implant mucositis or provide any specific inclusion criteria for disease [45]. The nature of the implant and the number of implant prostheses were not mentioned. One study also included patients with implant-supported overdenture. The nature of the comparator group (floss, interdental brush, mechanical tooth brushing) was different in the included. There are wide variations in the type of irrigants used with oral irrigators. In some studies, oral irrigators were used with tap water, while in others they used saline or chlorhexidine. The methods of assessing the clinical outcomes such as gingival inflammation and plaque score were measured using different indices and the results were reported with varying follow-up periods.

#### GRADE profile

A grade profile for systematic reviews with narrative results was undertaken using the new GradePro software (Supplementary Table 3). Due to wide heterogeneity in the studies, serious concerns about the risk of bias, inconsistency, indirectness, and imprecision were noted. The certainty of the evidence was very low for bleeding on probing, modified plaque index, and gingival index, and low for probing pocket depth. The Grade Summary of Findings table for systematic review gives reasons for downgrading the evidence is provided as Supplementary Table 3.

## Discussion

Peri-implant diseases (peri-implant mucositis and peri-implantitis) are caused by inflammation around the implant. This is due to the formation of microbial biofilm on the implant surface or peri-implant mucosa. Peri-implantitis results in severe bone loss and inflammation around the implant, loss of attachment, and pocket formation. Untreated peri-implant diseases often lead to the implant mobility, and loss of the implant [35, 36]. If diagnosed early and managed correctly, peri-implant mucositis can be managed effectively. Peri-implant diseases can be managed either via non-surgical periodontal therapy involving professional scaling and root planing or via surgery involving the use of bone graft or periodontal treatment [37]. Apart from professional treatment, patients’ education to maintain good oral hygiene around implants is vital to control the inflammation around the implant. Effective plaque control can be achieved by educating correct brushing technique to the patients along with the use of interdental aids [35, 38]. Interdental aids such as floss, oral irrigators, interdental brushes, and unitufted brushes are often prescribed to patients with dental implants. For effective management and prevention of peri‐implant diseases, clinicians need to consider their client’s personal preferences when recommending oral home care aids to achieve motivation, support compliance, and adherence to oral hygiene practices [8].Oral irrigators are known to be one of the easiest, most effective, and most patient-friendly tools for plaque control around implants. Oral delivers a powerful, pulsating stream of water or antimicrobial agent through a small nozzle or tip directed at areas between the teeth and the gum line. The water stream effectively loosens and flushes out food particles, plaque, and bacteria that can get trapped between teeth or in gum pockets. This significantly reduces the risk of plaque build-up and periodontal disease. The pulsating action also provides a powerful “hydromassaging” effect on the gums, effectively stimulating circulation and promoting gum health [39]. This allows for precise targeting of areas between teeth and along the gum line, making it easy to clean hard-to-reach spots. Oral irrigators are good adjuncts to toothbrushing for the mechanical removal of plaque, especially for people with braces, bridges, or other dental work [19–22]. Studies have demonstrated that oral irrigation effectively reduces periodontal and peri-implant inflammation, significantly reducing bleeding, gingival inflammation, clinical attachment loss, and probing depth. The reduction in gingival inflammation is better with oral irrigation compared to brushing alone. Few studies have even reported comparable effects of oral irrigators and interdental brushes for the control of biofilm [22, 24, 40, 46]. Our systematic review also noted that the GI, PI, and BOP reduction was better when oral irrigators were combined with mechanical tooth brushing. However, the certainty of evidence is low to support the superiority of oral irrigators over other interdental aids. Although the best results were noted when oral irrigators were combined with interdental brush and mechanical toothbrushing compared to the use of oral irrigators alone, the results were not statistically significant. We also found no difference in the clinical outcomes when oral irrigators are used alone or with an antimicrobial agent (chlorhexidine). Apart from reducing periodontal inflammation, only one study reported that oral irrigators can increase the thickness of the keratin layer around the implant, and no significant difference in the width of keratinized gingiva was noted with the use of oral irrigator or dental floss [41, 48]. Although previous animal and in-vitro studies have shown that oral irrigation can enhance bone formation around dental implants and increase the growth of the osteoblastic cells [42, 51, 52], we did not find any studies assessing the effect of oral irrigators on peri-implant bone levels. Hence, we cannot comment on the effect of oral irrigators in managing peri-implantitis, in terms of improvement in bone levels around implants. Moreover, the water from the oral irrigator does not reach the base of the pocket, the effect of oral irrigators in patients with deep pockets around implants is questionable. Studies have found that irrigation through a supragingival tip reached 44%–71% of pocket depth, with no difference with tip placement at 90° or 45° [53]. Subgingival tips delivered to 64%–100% of pocket depth, and were better than supragingival tips [54]. Hence, oral irrigators are still a better option compared to interdental brush and floss for patients who lack manual dexterity, elderly, physically and mentally challenged individuals, handicapped, and hospitalized patients. They are also ideal for patients with implant-supported dentures and overdentures as the flushing action of oral irrigators effectively removes microorganisms from the micro-irregularities of the prosthetic surfaces [45]. No statistically significant difference in improvement in quality of life and patient satisfaction in patients using mechanical toothbrushing alone and oral irrigator with regards to comfort or ease of use by patients with implants.

Additionally, we would like to highlight that the current evidence is based on a limited number of studies with only six months of follow-up. Additionally, in all the included studies comparing the use of oral irrigators on peri-implant inflammation, there was a significant heterogenicity in the comparator group used for assessing the efficacy of oral irrigators. While only one study has evaluated the additional benefit of using chlorhexidine over water for peri-implant tissues, previous studies comparing the use of oral irrigators with antimicrobial agents on periodontal tissues have shown better results than plain water. It is therefore essential that future studies compare the effect of different antimicrobial solutions and water in the maintenance of peri-implant health. No studies have assessed the effect of oral irrigators on the microbial profile, marginal bone levels, and the degree of osseointegration. Also, no clinical studies have compared the use of oral irrigators with different antimicrobial agents and different regimes. Based on the heterogenicity of the studies included, the certainty of evidence regarding the effects of oral irrigators on individual parameters of inflammation is low. Combining the overall effect of oral irrigators, we can conclude that the addition of oral irrigators to mechanical brushing would be beneficial in managing peri-implant disease by providing better plaque control and reducing bleeding sites. Future studies must assess the effect of oral irrigators on PD, CAL, and microbial profile around implants. Studies must also analyze and compare the nature and pressure of oral irrigation devices on the macro-design and micro-design of the implant surface and implant-supported prosthesis. Patients must be taught how to use the oral irrigator correctly to avoid damaging soft tissues and interdental papillae. Incorrect angulation and excessive water pressure can damage the junctional epithelium, dislodge the prosthesis, and lead to bacteremia in some patients. A pressure of 70–90 psi is considered safe for non-ulcerated attached gingiva, and 50–70 psi is safe for ulcerated oral soft tissues [39, 55].

## Conclusion

Peri-implant mucositis can be managed effectively by reinforcing good oral hygiene and plaque control. Oral irrigators are a good adjunct to mechanical toothbrushing for effectively removing microbial biofilm and controlling the inflammation around the implants. Based on the results of the included studies in this review, oral irrigation was found to be comparable to other interdental aids in managing peri-implant diseases. Oral irrigators when combined with mechanical toothbrushing are more effective in controlling inflammation and BOP compared to mechanical brushing alone. The decrease in inflammation, plaque scores, and BOP was noted irrespective of the nature of the solution used with an oral irrigator.

## Supplementary information

PRISMA Checklist

Supplementary Table 1 to Table 3

## Data Availability

Data will be available upon request.
