# Correction: Efficacy of oral irrigators compared to other interdental aids for managing peri-implant diseases: a systematic review

**DOI:** 10.1038/s41405-025-00312-0

**Published:** 2025-02-20

**Authors:** Gargi Gandhi, Bhoomika Sai Laxmi Masanam, Ananya Sudhakaran Nair, Nidhi Semani, Aditi Chopra, Venkitachalam Ramanarayanan

**Affiliations:** 1 Manipal College of Dental Sciences, Manipal, Manipal Academy of Higher Education, Manipal, Karnataka India; 2 Department of Periodontology, Manipal College of Dental Sciences, Manipal, Manipal Academy of Higher Education, Manipal, Karnataka India; 3 Department of Public Health Dentistry, Amrita School of Dentistry, Amrita Vishwa Vidyapeetham, Kochi, India

**Keywords:** Peri-implantitis, Dental implants

Correction to: *BDJ Open* 10.1038/s41405-025-00301-3, published online 29 January 2025

In this article the author’s name Gargi Gandhi was incorrectly written as Gargi Gandi. Second, the author’s name Venkitachalam Ramanarayanan was incorrectly written as Venkitachalam Ramanarayan. The authors apologie for any inconvenience caused. The original article has been corrected.

