# Supplementary material for: Efficacy of oral irrigators compared to other interdental aids for managing peri-implant diseases: a systematic review
Source: BDJ Open. 2025 Jan 29;11:7. doi: 10.1038/s41405-025-00301-3 (PMC11779913; doi:10.1038/s41405-025-00301-3)
Supplement: Supplementary file 2 — Supplementary Table 1 to Table 3 [file 41405_2025_301_MOESM2_ESM.docx]

| **Supplementary Table 1: List of excluded studies** | | |
| --- | --- | --- |
| **S/No.** | **Article details** | **Reason for exclusion** |
| 1. | Fassauer, H. M. (1975). Presentation of a portable mouth irrigator. Possibilities of improving the cleansing effect and the activation of the peripheral blood flow in periodontal prophylaxis and therapy. *Stomatologie der DDR*, *25*(11), 760-766. | Not a study on periimplantitis. Describes a new oral irrigator. |
| 2. | Barnes, C. M., Russell, C. M., Hlava, G. L., Utecht, B., & Reinhardt, R. A. (2003). A comparison of a waterpik dual-motor powered toothbrush and a manual toothbrush in affecting interproximal bleeding reduction and dental biofilm accumulation. *The Journal of Clinical Dentistry*, *14*(3), 49-52. | Not on periimplantitis |
| 3. | Zhang, J., McGrath, C., Chan, K. M., & Lam, O. L. (2022). A randomised clinical trial on the effect of oral antimicrobial sprays in institutionalised elders. *Gerodontology*, *39*(4), 391-400. | Not on periimplantitis; subjects with mild-to-moderate gingivitis. |
| 4. | Frascella, J. A., Fernández, P., Gilbert, R. D., & Cugini, M. (2000). A randomized, clinical evaluation of the safety and efficacy of a novel oral irrigator. *American Journal of Dentistry*, *13*(2), 55-58. | Not on periimplantitis; subjects with mild-to-moderate gingivitis. |
| 5. | Watts, E. A., & Newman, H. N. (1986). Clinical effects on chronic periodontitis of a simplified system of oral hygiene including subgingival pulsated jet irrigation with chlorhexidine. *Journal of Clinical Periodontology*, *13*(7), 666-670. | Patients with chronic periodontitis. |
| 6. | Abdellatif, H., Alnaeimi, N., Alruwais, H., Aldajan, R., & Hebbal, M. I. (2021). Comparison between water flosser and regular floss in the efficacy of plaque removal in patients after single use. *The Saudi Dental Journal*, *33*(5), 256-259. | Not on periimplantitis |
| 7. | Kalaga, A., Addy, M., & Hunter, B. (1989). Comparison of chlorhexidine delivery by mouthwash and spray on plaque accumulation. *Journal of periodontology*, *60*(3), 127-130. | Not on periimplantitis |
| 8. | Barnes, C. M., Russell, C. M., Reinhardt, R. A., Payne, J. B., & Lyle, D. M. (2005). Comparison of irrigation to floss as an adjunct to tooth brushing: effect on bleeding, gingivitis, and supragingival plaque. *Journal of Clinical Dentistry*, *16*(3), 71. | Not on periimplantitis |
| 9. | Sharma, N. C., Lyle, D. M., Qaqish, J. G., & Schuller, R. (2012). Comparison of two power interdental cleaning devices on plaque removal. *Journal of Clinical Dentistry*, *23*(1), 17. | Not in implants |
| 10. | Sharma, N. C., Lyle, D. M., Qaqish, J. G., & Schuller, R. (2012). Comparison of two power interdental cleaning devices on the reduction of gingivitis. *Journal of Clinical Dentistry*, *23*(1), 22. | Not on periimplantitis |
| 11. | Lyle, D. M., Goyal, C. R., Qaqish, J. G., & Schuller, R. (2016). Comparison of Water Flosser and Interdental Brush on Plaque Removal: A Single-Use Pilot Study. *The Journal of clinical dentistry*, *27*(1), 23-26. | Not on periimplantitis |
| 12. | Goyal, C. R., Lyle, D. M., Qaqish, J. G., & Schuller, R. (2016). Comparison of Water Flosser and Interdental Brush on Reduction of Gingival Bleeding and Plaque: A Randomized Controlled Pilot Study. *The Journal of Clinical Dentistry*, *27*(2), 61-65. | Not on periimplantitis |
| 13. | Eakle, W. S., Ford, C., & Boyd, R. L. (1986). Depth of penetration in periodontal pockets with oral irrigation. *Journal of clinical periodontology*, *13*(1), 39-44. | Not on periimplantitis |
| 14. | Moore, G. C., Smith, K. T., Christiansen, M. M., Anderson, L., Moravec, L. J., Okano, D. K., ... & Killeen, A. C. (2023). Effect of interproximal home oral hygiene on clinical parameters and inflammatory biomarkers in patients receiving periodontal maintenance. *Journal of periodontology*. | Not on periimplantitis |
| 15. | Southard, G. L., Parsons, L. G., Thomas, L. G., Woodall, I. R., & Jones, B. J. B. (1987). Effect of sanguinaria extract on development of plaque and gingivitis when supragingivally delivered as a manual rinse or under pressure in an oral irrigator. *Journal of Clinical Periodontology*, *14*(7), 377-380. | Not on periimplantitis |
| 16. | Pistorius, A., Willershausen, B., Steinmeier, E. M., & Kreisler, M. (2003). Efficacy of subgingival irrigation using herbal extracts on gingival inflammation. *Journal of periodontology*, *74*(5), 616-622. | Not on periimplantitis |
| 17. | Goyal, C. R., Lyle, D. M., Qaqish, J. G., & Schuller, R. (2015). Efficacy of Two Interdental Cleaning Devices on Clinical Signs of Inflammation: A Four-Week Randomized Controlled Trial. *The Journal of clinical dentistry*, *26*(2), 55-60. | Not on periimplantitis |
| 18. | Goyal, C. R., Lyle, D. M., Qaqish, J. G., & Schuller, R. (2013). Evaluation of the plaque removal efficacy of a water flosser compared to string floss in adults after a single use. *The Journal of clinical dentistry*, *24*(2), 37-42. | Not on periimplantitis |
| 19. | Goyal, C. R., Qaqish, J. G., Schuller, R., & Lyle, D. M. (2018). Evaluation of the Addition of a Water Flosser to Manual Brushing on Gingival Health. *The Journal of clinical dentistry*, *29*(4), 81-86. | Not on periimplantitis |
| 20. | Gallie, A. (2019). Home use of interdental cleaning devices and toothbrushing and their role in disease prevention. *Evidence-based dentistry*, *20*(4), 103-104. | Review paper |
| 21. | BATOOL, S. M., RASHEED, M., SYED, K., FAROOQ, A., AYUB, Z., & RAHEEM, A. Plaque Removal Efficacy of Dental floss Compare to Water Jet Following Single Use. *surfaces*, *8*, 9. | Not on periimplantitis |
| 22. | Goyal, C. R., Lyle, D. M., Qaqish, J. G., & Schuller, R. (2012). The addition of a water flosser to power tooth brushing: effect on bleeding, gingivitis, and plaque. *J Clin Dent*, *23*(2), 57-63. | Not on periimplantitis |
| 23. | Hoover, D. R., & Robinson, H. B. (1971). The comparative effectiveness of a pulsating oral irrigator as an adjunct in maintaining oral health. *Journal of periodontology*, *42*(1), 37-39. | Not on periimplantitis |
| 24. | Watt, D. L., Rosenfelder, C., & Sutton, C. D. (1993). The effect of oral irrigation with a magnetic water treatment device on plaque and calculus. *Journal of Clinical Periodontology*, *20*(5), 314-317. | Not on periimplantitis |
| 25. | Johnson, K. E., Sanders, J. J., Gellin, R. G., & Palesch, Y. Y. (1998). The effectiveness of a magnetized water oral irrigator (Hydro Fioss®) on plaque, calculus and gingival health. *Journal of clinical periodontology*, *25*(4), 316-321. | Not on periimplantitis |
| 26. | Ren, X., He, J., Cheng, R., Chen, Y., Xiang, Y., Zhang, Y., ... & Hu, T. (2023). The Efficacy and Safety of Oral Irrigator on the Control of Dental Plaque and Gingivitis: A Randomized, Single-Blind, Parallel-Group Clinical Trial. *International Journal of Environmental Research and Public Health*, *20*(4), 3726. | Not on periimplantitis |
| 27. | Corbella, S., Del Fabbro, M., Taschieri, S., De Siena, F., & Francetti, L. (2011). Clinical evaluation of an implant maintenance protocol for the prevention of peri‐implant diseases in patients treated with immediately loaded full‐arch rehabilitations. *International Journal of Dental Hygiene*, *9*(3), 216-222. | No oral irrigator used |
| 28. | Charalampakis, G., Rabe, P., Leonhardt, Å., & Dahlén, G. (2011). A follow‐up study of peri‐implantitis cases after treatment. *Journal of clinical periodontology*, *38*(9), 864-871. | No oral irrigator used |
| 29. | Froum, S. J., Froum, S. H., & Rosen, P. S. (2012). Successful management of peri-implantitis with a regenerative approach: a consecutive series of 51 treated implants with 3-to 7.5-year follow-up. *International Journal of Periodontics and Restorative Dentistry*, *32*(1), 11. | No oral irrigator used |
| 30. | Sahm, N., Becker, J., Santel, T., & Schwarz, F. (2011). Non‐surgical treatment of peri‐implantitis using an air‐abrasive device or mechanical debridement and local application of chlorhexidine: a prospective, randomized, controlled clinical study. *Journal of clinical periodontology*, *38*(9), 872-878. | Irrigation is done just before treatment |
| 31. | Renvert, S., Samuelsson, E., Lindahl, C., & Persson, G. R. (2009). Mechanical non‐surgical treatment of peri‐implantitis: a double‐blind randomized longitudinal clinical study. I: clinical results. *Journal of clinical periodontology*, *36*(7), 604-609. | No oral irrigator used |
| 32. | Lee, W., & Park, J. B. (2023). The clinical effect of sodium hypochlorite oral rinse on peri-implantitis lesion: A pilot study. *Heliyon*, *9*(5). | No oral irrigator used |
| 33. | Ciancio, S. G., Lauciello, F., Shibly, O., Vitello, M., & Mather, M. (1995). The effect of an antiseptic mouthrinse on implant maintenance: plaque and peri‐implant gingival tissues. *Journal of periodontology*, *66*(11), 962-965. | No oral irrigator used |
| 34. | De Siena, F., Francetti, L., Corbella, S., Taschieri, S., & Del Fabbro, M. (2013). Topical application of 1% chlorhexidine gel versus 0.2% mouthwash in the treatment of peri‐implant mucositis. A n observational study. *International Journal of Dental Hygiene*, *11*(1), 41-47. | No oral irrigator used |
| 35. | Hallström, H., Lindgren, S., & Twetman, S. (2017). Effect of a chlorhexidine‐containing brush‐on gel on peri‐implant mucositis. *International Journal of Dental Hygiene*, *15*(2), 149-153. | No oral irrigator used |
| 36. | Wolff, L., Kim, A., Nunn, M., Bakdash, B., & Hinrichs, J. (1998). Effectiveness of a sonic toothbrush in maintenance of dental implants: A prospective study. *Journal of clinical periodontology*, *25*(10), 821-828. | No oral irrigator used |
| 37. | Lambert, T. J. (2022). A Home Care Regimen With Oral-B iO Toothbrush and Targeted Clean Brush Head to Reduce Peri-Implant Mucositis. *Compendium of Continuing Education in Dentistry (Jamesburg, NJ: 1995)*, *43*(3), f5-fE13. | There are no results mentioned |
| 38. | Hentenaar, D. F., De Waal, Y. C., Van Winkelhoff, A. J., Meijer, H. J., & Raghoebar, G. M. (2020). Non‐surgical peri‐implantitis treatment using a pocket irrigator device; clinical, microbiological, radiographical and patient‐centred outcomes—A pilot study. *International Journal of Dental Hygiene*, *18*(4), 403-412. | There are no comparators |
| 39. | Jervøe‐Storm, P. M., Hablützel, A. S., Bartels, P., Kraus, D., Jepsen, S., & Enkling, N. (2021). Comparison of irrigation protocols for the internal decontamination of dental implants—results of in vitro and in vivo studies. *Clinical Oral Implants Research*, *32*(10), 1168-1175. | No oral irrigator used |
| 40. | Felo, A., Shibly, O., Ciancio, S. G., Lauciello, F. R., & Ho, A. (1997). Effects of subgingival chlorhexidine irrigation on peri-implant maintenance. *American journal of dentistry*, *10*(2), 107-110. | Not found |
| 41. | Caccianiga, G., Rey, G., Caccianiga, P., Leonida, A., Baldoni, M., Baldoni, A., & Ceraulo, S. (2021). Rough Dental Implant Surfaces and Peri-Implantitis: Role of Phase-Contrast Microscopy, Laser Protocols, and Modified Home Oral Hygiene in Maintenance. A 10-Year Retrospective Study. *Applied Sciences*, *11*(11), 4985. | Comparator group not relevant; (no other oral hygiene method.) 2 types of implants surfaces are being compared in this study to analyse the number of implants lost due to periimplantitis. |
| 42. | Porras, R., Anderson, G. B., Caffesse, R., Narendran, S., & Trejo, P. M. (2002). Clinical response to 2 different therapeutic regimens to treat peri‐implant mucositis. *Journal of periodontology*, *73*(10), 1118-1125. | No oral irrigator used |

**Supplementary Table 2: Risk of bias analysis for all the included study**

| **Study (author and year)** | **Bias arising from the randomization process** | **Bias due to deviation from intended information** | **Bias arising due to missing data** | **bias in the measurement of the outcome in the included studies** | **Bias in the selection of reports results** | **Overall** |
| --- | --- | --- | --- | --- | --- | --- |
| **Magnuson et al 2013** | Low | Low | Some concerns | Low | Some concerns | Some concerns |
| **Salles et al. 2021** | Low | low | Some concerns | Low | Low | Low |
| **Bunk et al. 2020** | low | low | Some concerns | Low | Low | Low |
| **Olimov et al 2020** | High | High | High | Some concerns | Some concerns | High |
| **Sgarbanti et al. 2021** | High | High | Some concerns | Low | Some concerns | High |
| **Salles et al. 2021** | Low | low | Some concerns | Low | Low | Low |
| **Tütüncüoğlu et al 2021** | Low | Low | Low | Low | Low | Low |

**Supplementary Table 3: Summary of findings tables (GRADE analysis)**

| **No of studies** | **Certainty assessment** | | | | | | | | **Findings** | **Certainty or strength of evidence** | **Importance** |
| --- | --- | --- | --- | --- | --- | --- | --- | --- | --- | --- | --- |
|  | **Study design** | **Interventions/**  **Comparator (n- no of participant)** | **Risk of bias** | **Inconsistency** | | **Indirectness** | **Imprecision** | **Other considerations** |  |  |  |
| **Bleeding on probing** | | | | | | | | | |  |  |
| 5 | Randomized trials | Toothbrushing alone (N=73)  Interdental brush + toothbrushing (N=45)  Oral irrigators+ toothbrushing (N=100)  Interdental brush + oral irrigator +  Toothbrushing (0)  Floss + Toothbrushing (N=25) | Serious ^a^ | Serious ^b^ | | Serious ^c^ | Serious ^d^ | None | There was a significant reduction in BOP for the oral irrigators group in Bunk 2020 (12 weeks), Magnuson 2013 (4 weeks), and Tütüncüoğlu 2021 (4 weeks). Tütüncüoğlu 2021 showed a significant difference only in the 4th week but not in the 2nd and 12th weeks. Time points were different across studies and method of measuring BOP was different. Hence meta-analysis was not performed. | ⨁◯◯◯ Very low | IMPORTANT |
| **Modified plaque index** | | | | |  |  |  |  |  |  |  |
| 4 | Randomized trials | Toothbrushing alone (N=73)  Interdental brush + toothbrushing (N=15)  Oral irrigators+ toothbrushing (N=85)  Interdental brush + oral irrigator +  Toothbrushing (N=0)  Floss + Toothbrushing (N=12) | Serious ^e^ | not serious | | Serious ^c^ | Serious ^d^ | None | No significant differences in mPI were reported in the included studies (Bunk 2020, Salles 2021, Sgarbanti 2020, Tütüncüoğlu 2021) | ⨁◯◯◯ Very low | IMPORTANT |
| **Probing Pocket Depth** | | | | |  |  |  |  |  |  |  |
| 4 | Randomized trials | Toothbrushing alone (N=73)  Interdental brush + toothbrushing (N=15)  Oral irrigators+ toothbrushing (N=85)  Interdental brush + oral irrigator +  Toothbrushing (N=0)  Floss + Toothbrushing (N=12) | not serious | not serious | | Serious ^c^ | Serious ^d^ | none | No significant differences in probing pocket depth were reported in three studies  Salles 2021  Sgarbanti 2021  Tütüncüoğlu 2021  No information on probing pocket depth in Bunk 2020 | ⨁⨁◯◯ Low | CRITICAL |
| **Gingival Index (assessed with Gingival index and modified Gingival index)** | | | | |  |  |  |  |  |  |  |
| 3 | randomized trials | Toothbrushing alone (N=66)  Interdental brush + toothbrushing (N=45)  Oral irrigators+ toothbrushing (N=55)  Interdental brush + oral irrigator +  Toothbrushing (N=45)  Floss + Toothbrushing (N=0) | Serious | Serious ^f^ | | Serious ^g^ | Serious ^d^ | none | Bunk 2020: No change in mucosal severity scores between the groups  Salles 2021: Non-significant difference between the groups  Olimov et al 2020: Baseline values not reported. Combined the use of an oral irrigator with an interdental brush and toothbrushing. Hence of effect of oral irrigator cannot be assessed. | ⨁◯◯◯ Very low | IMPORTANT |

#### Explanations

a. Two studies have a high risk of bias (Olimov et al 2020 and Sgarbanti et al 2021) which might affect the results.

b. A variation was noted in the outcomes. Three studies showed significantly lower BOP for the oral irrigation group while two studies did not show a significant difference.

c. Indirectness is present as there are differences in the study population (few studies are on clinically diagnosed peri-implantitis while others are on patients with implants; no mention of peri-implantitis). There are also differences in intervention. Some studies have used oral irrigators in conjunction with mechanical toothbrushes while others are with interdental brushes or a combination of all three. No indirectness as far as outcome assessment is concerned (PD). Time differences in outcome;

d. Optimal Information Size not met. Sample sizes are small (total < 400). The confidence interval overlaps with no effect. One study (Sgarbanti et al 2021) has a high risk of bias;

f. There are inconsistencies in the effect of bleeding on probing. One study (Olimov et al. 2020) reports a significant difference between groups while Salles et al 2021 reports no significant differences;

g. Indirectness is present as there are differences in the study population (few studies are on clinically diagnosed peri-implantitis while others are on patients with implants; no mention of peri-implantitis). There are also differences in intervention. Some studies have used oral irrigators in conjunction with mechanical toothbrushes while others are with interdental brushes or a combination of all three. The timepoint differences in outcome. In one study (Bunk et al. 2020), BOP is measured using the Mucosal severity score.
